# Qing Fei Hua Xian Decoction ameliorates bleomycin-induced pulmonary fibrosis by suppressing oxidative stress through balancing ACE-AngII-AT1R/ACE2-Ang-(1-7)-Mas axis

**DOI:** 10.22038/IJBMS.2022.67042.14700

**Published:** 2023-01

**Authors:** Rui-jie Li, Chao-yan Wu, Hao-liang Ke, Xiu-ping Wang, Ying-wen Zhang

**Affiliations:** 1 Department of Integrated Chinese and Western Medicine, Zhongnan Hospital of Wuhan University, Wuhan 430071, Hubei, China; 2 School of Traditional Chinese Medicine, Hubei University of Chinese Medicine, Wuhan 430065, Hubei, China

**Keywords:** Extracellular matrix, Lung diseases, NADPH oxidases, Oxidative stress, Pulmonary fibrosis, Renin-angiotensin system

## Abstract

**Objective(s):**

We aimed to investigate the preventative effect of Qing Fei Hua Xian Decoction (QFHXD) against pulmonary fibrosis (PF) and its potential mechanisms.

**Materials and Methods:**

Bleomycin (BLM)-induced rats were respectively treated with 413.3, 826.6, and 1239.9 mg/kg of QFHXD and prednisone for 28 days. The lung tissues of rats were collected on day 28 for histological and western blotting analysis.

**Results:**

QFHXD significantly reduced alveolus inflammation, collagen accumulation, and fibrosis deposition in BLM-induced PF rats (*P*<0.05). Collagen I and III, vimentin, and α-smooth muscle actin(α-SMA) expression levels were likewise decreased in PF rats treated with QFHXD (*P*<0.05). Additionally, QFHXD increased the expression of nuclear factor erythroid 2-related factor 2 (Nrf2) while decreasing NADPH oxidase 4 (NOX4) expression (*P*<0.05). Furthermore, QFHXD suppressed the PF progression by down-regulating Angiotensin-Converting Enzyme (ACE) -Angiotensin II (AngII) -Angiotensin II Type 1 Receptor (AT1R) axis (*P*<0.01) and up-regulating Angiotensin-Converting Enzyme 2 (ACE2) -Angiotensin-(1-7) (Ang-(1-7)) -Mas axis (*P*<0.05).

**Conclusion:**

QFHXD suppressed inflammatory infiltration and PF brought on by BLM in lung tissues through reducing oxidative stress by maintaining the equilibrium of ACE-AngII-AT1R and ACE2-Ang-(1-7) -Mas axes. This study may provide a novel clinical therapy option for PF.

## Introduction

Pulmonary fibrosis (PF) is a progressive, fatal, and chronic disorder marked by inflammatory infiltration of the lungs and fibrosis of the lung parenchyma (1). Patients with PF gradually lose lung function as the disease worsens, which may lead to respiratory failure or even death (2). To date, only pirfenidone and nintedanib, and lung transplantation have been proven to be effective treatments for PF globally (3), and no drugs have shown a predicted survival advantage for PF. Therefore, novel medicines are urgently required to slow the progression of PF and improve the quality of life for PF patients.

Oxidative stress is associated with reactive oxygen species (ROS) and reactive nitrogen species (RNS) overproduction, leading to oxidation/anti-oxidation disequilibrium (4). Idiopathic pulmonary fibrosis (IPF) progresses due to an excess of ROS and activation of multiple NADPH oxidase (NOX) isoforms (5). Elevated levels of ROS induce PF through pathological processes, such as alveolar epithelial cell (AEC) apoptosis, inflammatory cell infiltration, collagen accumulation, and epithelial-mesenchymal transition (EMT) (6). NADPH oxidase 4 (NOX4), one of the NOX family oxidoreductases, is crucial for PF development by inducing intracellular ROS generation, AEC death, Smad phosphorylation, and extracellular matrix (ECM) production (7, 8). Numb expression is suppressed by nuclear factor erythroid 2-related factor 2 (Nrf2) to affect EMT-mediated PF via anti-oxidant pathway (9), and deficits of Nrf2 are associated with the onset of PF. Moreover, NOX4-Nrf2 imbalance has been discovered in the lung tissue of PF patients, and this suggests that a treatment approach aimed at restoring the NOX4-Nrf2 redox equilibrium in PF might be effective (10).

Previous investigations have indicated that an activated pulmonary renin-angiotensin system (RAS) is linked to initiation and progression of PF. Substantial evidence indicated that up-regulating the angiotensin (Ang)-converting enzyme (ACE)/AngII/ angiotensin II type 1 receptor(AT1R) axis could exacerbate PF, and that activation of angiotensin-converting enzyme 2 (ACE2) and generation of enzymatic product Ang-(1-7) could modify the intrapulmonary component of RAS (11). The ACE2-Ang-(1-7)-Mas axis, counteracting ACE-AngII-AT1R axis activity, protects against PF (12). Additionally, AngII induces collagen deposition and fibrosis via activation of the NOX4/ROS/RhoA/Rock pathway. Besides, by lowering NOX4 and ROS generation, the ACE2-Ang-(1-7)-Mas axis could prevent collagen deposition and PF caused by bleomycin (BLM) or AngII. Thus, ACE2-Ang-(1-7)-Mas axis displays a critical anti-oxidant function in NOX4-derived ROS-mediated PF (13). Due to the close relationship between ACE-AngII-AT1R/ACE2-Ang-(1-7)-Mas axis and oxidative stress, maintaining a balance between the two may help to limit the development of PF. 

Qing Fei Hua Xian Decoction (QFHXD) was constructed to treat PF based on Chinese medical theory and clinical experience related to lung diseases. QFHXD is composed of 14 herbs, namely *Astragalus membranaceus*, *Angelica sinensis*, *Ephedra*, *Amygdalin, Pinellia*, *Whole trichosanthes kirilowii*, *Radix pseudostellariae, Semen lepidii, Radix paeoniae rubra, *and *Thunberg fritillary bulb,* etc. A number of these herbal drugs have been well-documented to exhibit significant anti-fibrosis and anti-oxidant properties. For instance, Angelica sinensis polysaccharide has been found to inhibit the EMT progression of IPF by down-regulating the expression of differentiation antagonizing non-protein coding RNA (DANCR) and suppressing AU-binding factor 1 (AUF1)-mediated FOXO3 translation(14). Amygdalin inhibited the transforming growth factor-β1 (TGF-β1) expression and suppressed small mothers against decapentaplegic (Smad)2/3 phosphorylation to slow down the EMT process (15). Heterophylline B extracted from Radix pseudostellariae inhibited BLM-induced PF, possibly by down-regulating TGF-Smad2/3 and adenosine 5-monophosphate-activated protein kinase (AMPK)-mediated stimulator of interferon genes (STING) signaling pathways (16). Total paeony glucosides isolated from the roots of Radix paeoniae rubra have been shown to protect the anti-oxidant defense system against oxidative stress-induced diseases (17). However, the underlying mechanisms of QFHXD against PF remain unclear.

In this study, we hypothesized that QFHXD could play a protective role in PF by attenuating oxidative stress, possibly by modulating ACE/AngII/AT1R and ACE2/Ang-(1-7)/Mas axes, given the link between PF pathogenesis and oxidative stress and the ACE-AngII-AT1R/ACE2-Ang1-7-Mas axis. Here, BLM-induced PF rat models were used to assess the preventative effects of QFHXD against oxidative stress and PF. Moreover, the influence of QFHXD on BLM-induced PF development *in vivo* and the anti-oxidative actions of QFHXD through restoring ACE-AngII-AT1R/ACE2-Ang1-7-Mas axis homeostasis were clarified. The findings in this study would provide theoretical support for the clinical treatment of PF. 

## Materials and Methods

***Herbal medicines and reagents***

Jingpai Chizheng Tang Pharmaceutical Co. LTD. (Hubei, China) supplied QFHXD, containing *Astragalus membranaceus *(Huangqi),* Angelica sinensis *(Danggui),* Ephedra *(Mahuang),* Amygdalin *(Xinren),* Pinellia *(Ban Xia),* Whole trichosanthes kirilowii *(Gulou),* Radix pseudostellariae *(Taizishen),* Semen lepidii *(Tinglizi),* Radix paeoniae rubra *(Chishao),* Areca-nut *(Binglang),* Immature bitter orange *(Zhishi)*, Thunberg fritillary bulb *(Zhebeimu),* Loofah sponge *(Sigualuo), and* Liquorice *(Gancio). All herbal medicines were water-extracted and subsequently dried. Prednisone tablets were acquired from the Xianju Pharmaceutical Co. LTD. (Zhejiang, China). BLM (lot: Y0125051) was given by Yeasen Biotechnology Co., LTD. (Shanghai, China). 

***Animals***

 Two-month SD male rats (200–220 g) were bought from Hunan SJA Laboratory Animal Co., LTD. (license No. SCXK(X)2019-0004; laboratory animal quality certification: No.430727211102922634). This study was authorized by Zhongnan Hospital Affiliated with Wuhan University’s Experimental Animal Welfare Ethics Committee (Approval No. ZN2021068). Animal treatments were conducted according to the National Institutes of Health’s Guide for the Care and Use of Laboratory Animals (NIH Publications No. 8023, revised 1978) and Wuhan University’s policy for the protection and use of experimental animals.

***BLM-induced PF rat models and drug administration***

Using a random number generator, 60 male SD rats were assigned to six groups (n = 10 in each): control, fibrosis, prednisone, QFHXD-413.3 mg/kg, QFHXD-826.6 mg/kg, and QFHXD-1239.9 mg/kg. In the fibrosis, prednisone, and QFHXD groups, PF rats were generated by intraperitoneally injecting 1.5% isoflurane anesthesia and then receiving 5 mg/kg of BLM with sterile 0.9% saline endotracheally. The control rats received the same amount of sterile saline. For Animal Equivalent Dose (AED, mg/kg), we multiplied the human dosage (mg/kg) by the K_m_ ratio. QFHXD is administered in doses of 4,000 mg/60 kg to adults according to the drug instruction, while prednisone is in doses of 10 mg/60 kg to adults. On day 1 after intratracheal injection, QFHXD-413.3 mg/kg, -826.6 mg/kg, and -1239.9 mg/kg groups received intragastric administration of 413.3, 826.6, and 1239.9 mg/kg of QFHXD, respectively, and the prednisone group received 0.25 mg/kg of prednisone daily for 28 consecutive days. Intragastrical injections of normal saline were administered to both the control and fibrosis groups. For further research, all rats were slaughtered on day 28 and their lung tissues were collected. 

***Hematoxylin and eosin (H&E) and masson’s trichrome (MT) staining***

Following fixation with 4% paraformaldehyde for 24 hr, dehydration, and embedding in paraffin, sections of the lungs (5-µm thickness) were processed for H&E and MT staining. The stained lung tissue sections were observed under an upright fluorescence microscope (200 × magnification, Olympus, Tokyo, Japan). The Alveolitis score was used to semi-quantitatively assess histological alterations in lung tissues as a result of inflammation of the alveoli(18). Alveolitis was assessed using H&E-stained sections based on the following criteria: none (0): absence of alveolitis; mild (1+): an infiltrate of mononuclear cells thickening the alveolar septum; involvement confined to focal, basal pleural lesions covering ≤ 20 % of the lung area, well preserved alveolar architecture; moderate (2+): Alveolitis that covers 20–50% of the lung, with a pleural emphasis; severe (3+): Diffuse alveolitis affecting half of the lungs, with occasional solid mononuclear cells in the alveoli, and interstitial and/or alveolar bleeding. Based on the ratio of pulmonary collagen-positive in MT staining (blue) in histological sections, Image J software (National Institutes of Health, MD, USA) was used to quantify PF. 

***Western blotting analysis***

Total proteins were extracted from lung tissues by homogenizing them in ice-cold radioimmunoprecipitation lysis buffer (Beyotime, Shanghai, China) with a phosphorylated protease inhibitor (AS1008, ASPEN, South Africa). A BCA protein concentration assay kit (AS1086, ASPEN, South Africa) was utilized to measure the protein concentration. Thereafter, SDS-PAGE (7.5-12%) was applied to separate the same quantity of protein (20 μg), followed by transferring the proteins to polyvinylidene difluoride membranes (Millipore, MA, USA). Membranes were blocked with non-fat dried milk (5%) at 25 °C for 60 min, and next incubated at 4 °C overnight with the following primary antibodies: anti-ACE (Rabbit, ab254222, Abcam, Cambridge, UK); anti-Angiotensinogen(AGT)(Rabbit, A11689, ABclonal, MA, USA); anti-AT1R (Rabbit, ab124734, Abcam); anti-ACE2 (Rabbit, ab108252, Abcam); anti-Mas (Rabbit, DF2818, AFF biotech); anti-α-smooth muscle actin(α-SMA)(Rabbit, 14395-1-AP, Proteintech, Wuhan, China); anti-Vimentin (Rabbit, 10366-1-AP, Proteintech); anti-Collagen-I (Mouse, 66761-1-Ig, Proteintech); anti-Collagen-III (Rabbit, 22734-1-AP, Proteintech); anti-Nrf2 (Rabbit, #33649, CST, MA, USA); anti-NOX4 (Rabbit, 14347-1-AP, Mitaka, Wuhan, China) and anti-β-actin (Rabbit, TDY051, TDY Biotech Co., LTD, Beijing, China). Thereafter, membranes were rinsed three times with TBS-T at 5-minute intervals, followed by an incubation of 30 min with goat anti-rabbit/anti-rat IgG labeled with horseradish peroxidase (1:10000, Biosynthesis Biotechnology Co., LTD., Beijing, China). TBS-T was rinsing the membranes every 5 min for 4 times. A prepared enhanced chemiluminescence kit (GE Healthcare, IL, USA) was used to produce chemiluminescent signals, and normalizing the ratio of targeted protein to β-actin was performed. The targeted band gray values were addressed and quantified with AlphaEaseFC 4.0 software (Alpha Innotech, CA, USA). 

***Statistical analysis***

The values were mean + standard deviation (SD). One-way ANOVA was conducted for statistical analysis using Prism 9.0 (GraphPad Software, CA, USA). The results were deemed statistically different when *P*<0.05.

## Results

***QFHXD ameliorated alveolus inflammation and BLM-induced PF***

**Figure 1:**
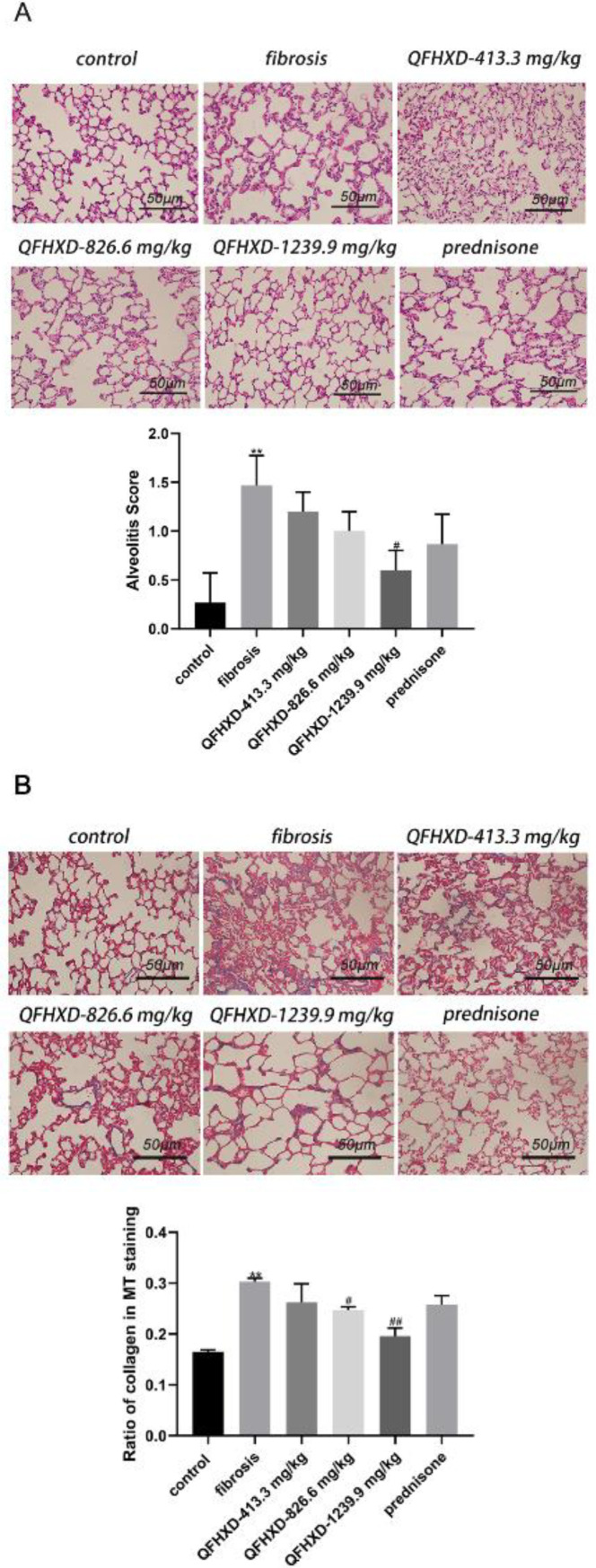
Qing Fei Hua Xian Decoction reduced Bleomycin-induced alveolus inflammation and pulmonary fibrosis in rats. (A) Representative images of Hematoxylin and Eosin staining of lung tissues, magnification × 200. The scale bar was 50 μm. Alveolitis score in each group was evaluated based on Hematoxylin and Eosin staining images. (B) Representative images of Masson’s trichrome staining of lung tissues, magnification × 200. The scale bar was 50 μm. Collagen ratios of the different groups were quantified by using Image J software. Data were expressed as mean ± SD (n = 3). **P<0.01 vs control group; ##*P*<0.01, #*P*<0.05 vs fibrosis group

As shown in Figure 1A, lung tissues exhibited histological changes. Within the control group, there was an integral lung structure and normal alveolar septum, whereas the fibrosis group indicated thickened and widened alveolar septum, collapsed alveolus fusion, and a significant number of inflammatory cell infiltrations. Furthermore, in the fibrosis group, alveolitis was considerably more severe compared with the control group (*P*<0.01). Clearly, QFHXD administration improved alveolitis, particularly in the QFHXD-1239.9 mg/kg group (vs fibrosis group, *P*<0.05). 

Similarly, in the control group, MT staining revealed a few blue collagen fibers in the lung tissues. In contrast, in the fibrosis group, there were many blue collagen fibers, and the collagen ratio was significantly higher (*P*<0.01). QFHXD administration considerably decreased the collagen ratio (QFHXD-826.6 mg/kg and QFHXD-1239.9 mg/kg vs fibrosis group, *P*<0.05 and *P*<0.01, respectively, Figure 1B). 

***QFHXD suppressed the expression of collagens and marker proteins***

**Figure 2:**
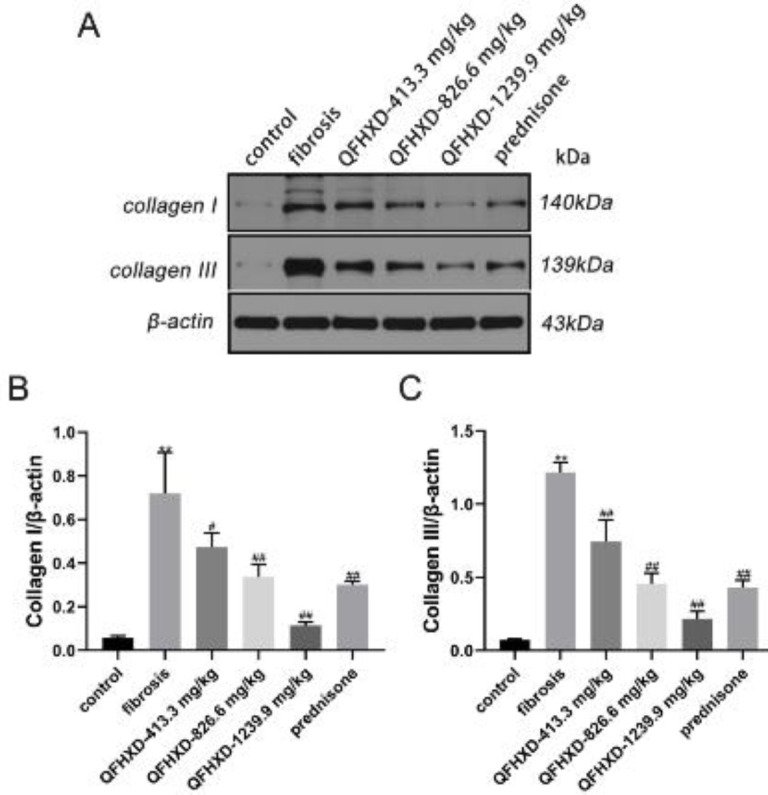
Qing Fei Hua Xian Decoction suppressed collagen I and III expressions in lung tissues of Bleomycin-induced rats. (A-C) Protein expressions of collagen I and III with western blotting analysis. Values were expressed as mean ± SD (n = 3). **P<0.01 vs control group; ##*P*<0.01, #*P*<0.05 vs fibrosis group

The protein expressions of representative collagen, such as collagen I, and collagen III (Figure 2A), were analyzed to determine the effect of QFHXD on collagen deposition. In contrast with the control group, collagen I expression was up-regulated in the BLM-induced fibrosis group (*P*<0.01, Figure 2B), while QFHXD (particularly in QFHXD-826.6 mg/kg and QFHXD-1239.9 mg/kg groups) and prednisone administration down-regulated the protein expression of collagen I in PF rats (*P*<0.01). Similarly, the collagen III protein expression was higher in the fibrosis group than in the control group (*P*<0.01), while all treatment groups decreased the collagen III protein expression dramatically (vs fibrosis group, *P*<0.01, Figure 2C).

**Figure 3:**
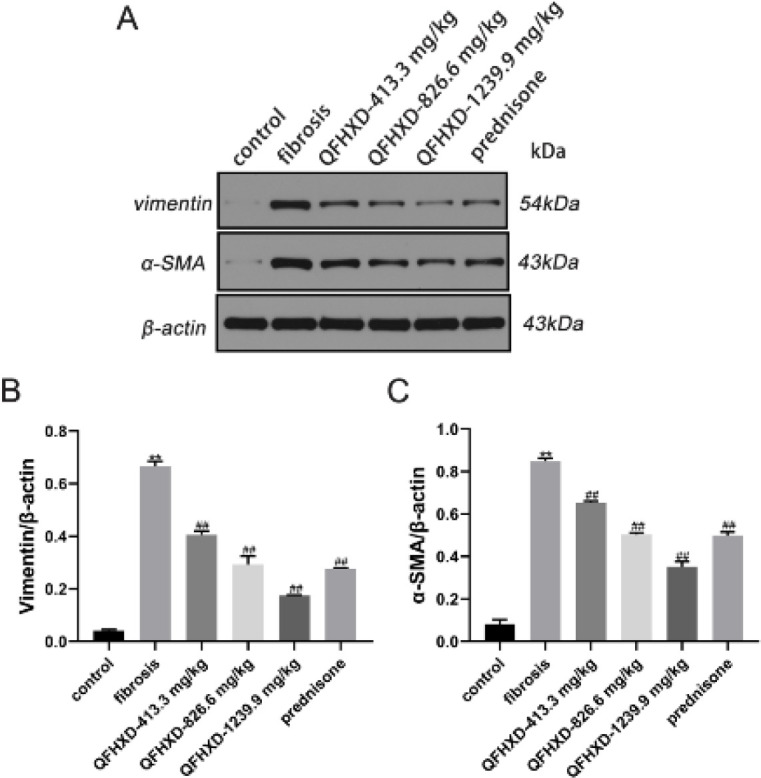
Qing Fei Hua Xian Decoction down-regulated vimentin and α-smooth muscle actin expressions in lung tissues of Bleomycin-induced rats. (A-C) protein expressions of vimentin and α-smooth muscle actin with western blotting analysis. Values were expressed as mean ± SD (n = 3). **P<0.01 vs control group; ##*P*<0.01, #*P*<0.05 vs fibrosis group

The expressions of vimentin and α-SMA, which indicate the progression of PF(19), are in Figure 3A. In BLM-induced rats, BLM treatment markedly increased the expression of vimentin and α-SMA (vs control group, *P*<0.01), whereas QFHXD and prednisone treatment decreased the expression of these proteins (*P*<0.01, Figure 3B and 3C).

***QFHXD down-regulated ACE-AngII-AT1R and up-regulated ACE2-Ang-(1-7)-Mas axis***

**Figure 4.**
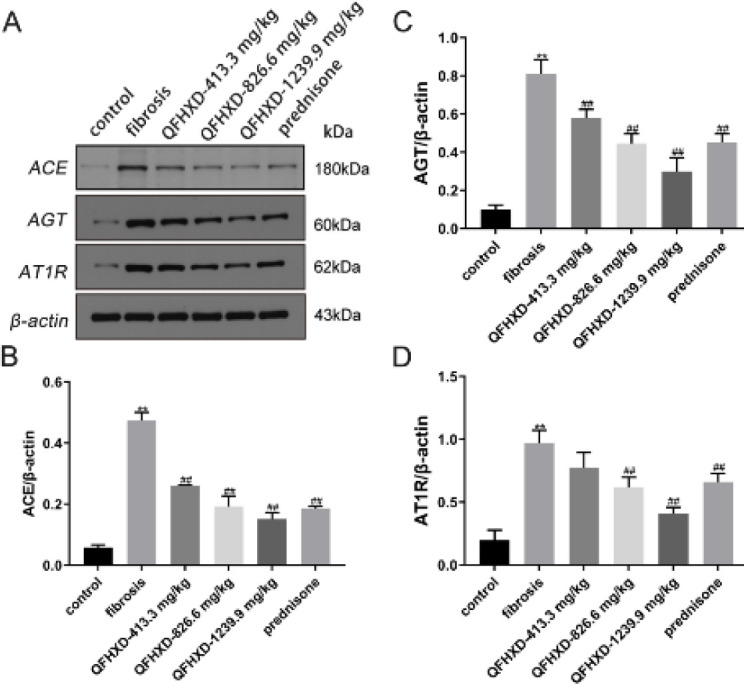
Qing Fei Hua Xian Decoction down-regulated angiotensin-converting enzyme, angiotensinogen, and angiotensin II type 1 receptor protein expressions in lung tissues of Bleomycin-induced rats. (A-D) Protein expressions of angiotensin-converting enzyme, angiotensinogen, and angiotensin II type 1 receptor with western blotting analysis. Values were expressed as mean ± SD (n = 3). **P<0.01 vs control group; ##*P*<0.01, #*P*<0.05 vs fibrosis group

AGT is the only substrate for all Ang peptides. It has been reported that AGT is up-regulated in the AEC of BLM-induced rats (20). Herein, the protein expressions of ACE, AGT, and AT1R were shown in Figure 4A. BLM induction evidently increased the levels of ACE, AGT, and AT1R proteins (vs control group, *P*<0.01). Nevertheless, QFHXD and prednisone decreased ACE (Figure 4B), AGT (Figure 4C), and AT1R (Figure 4D) protein expressions (vs fibrosis group, *P*<0.01). Between QFHXD-413.3 mg/kg and the fibrosis group, there was no difference in AT1R protein expression.

**Figure 5:**
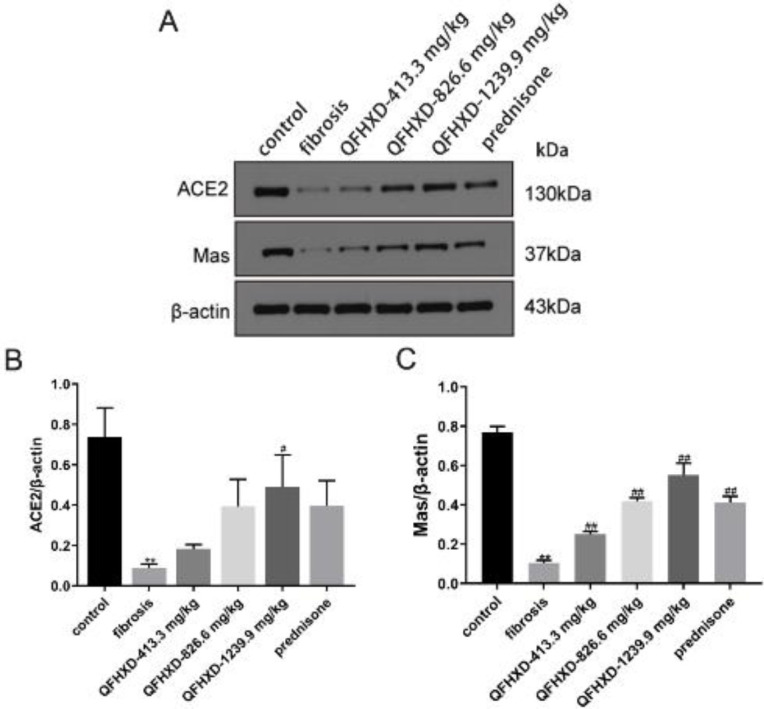
Qing Fei Hua Xian Decoction up-regulated angiotensin-converting enzyme 2 and Mas protein expressions in lung tissues of Bleomycin-induced rats. (A-C)The protein expressions of angiotensin-converting enzyme 2 and Mas with western blotting analysis. Values were expressed as mean ± SD (n = 3). **P<0.01 vs control group; ##*P*<0.01, #*P*<0.05 vs fibrosis group

As can be seen in Figure 5A, ACE2 and Mas were expressed. Instead, the expression of ACE2 protein was down-regulated in the fibrosis group (vs control group, *P*<0.01), while increasing in the QFHXD-1239.9 mg/kg group (*P*<0.05, Figure 5B). Similarly, Mas protein level was also obviously down-regulated in the BLM-induced fibrosis group (vs control group, *P*<0.01). However, QFHXD and prednisone intervention considerably up-regulated the Mas expression in the BLM-induced rats (*P*<0.01, Figure 5C). 

***QFHXD reduced the overexpression of NOX4 and promoted the expression of Nrf2***

**Figure 6:**
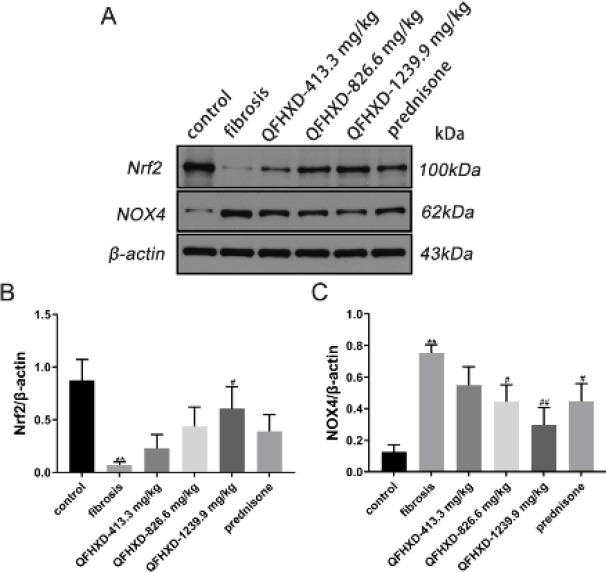
Qing Fei Hua Xian Decoction inhabited NADPH oxidase 4 and facilitated nuclear factor erythroid 2-related factor 2 protein expression in lung tissues of Bleomycin-induced rats. (A-C) Protein expressions of nuclear factor erythroid 2-related factor 2 and NADPH oxidase 4 with western blotting analysis. Values were expressed as mean ± SD (n= 3). **P<0.01 vs control group; ##*P*<0.01, #*P*<0.05 vs fibrosis group

The protein expressions of NOX4 and Nrf2 were indicated in Figure 6A. As a result of BLM stimulation, there was a dramatic decrease in Nrf2 protein value in the fibrosis lung tissues (vs control group, *P*<0.01), while the QFHXD-1239.9 mg/kg group markedly up-regulated the Nrf2 protein expression (vs fibrosis group, *P*<0.05*,* Figure 6B). In contrast, BLM induction up-regulated the expression of NOX4 protein in the control tissues (*P*<0.01). Apart from the QFHXD-413.3 mg/kg group, all treatment groups showed significant down-regulation of NOX4 protein overexpression (*P*<0.05, Figure 6C), particularly in the QFHXD-1239.9 mg/kg group (*P*<0.01). 

**Figure 7:**
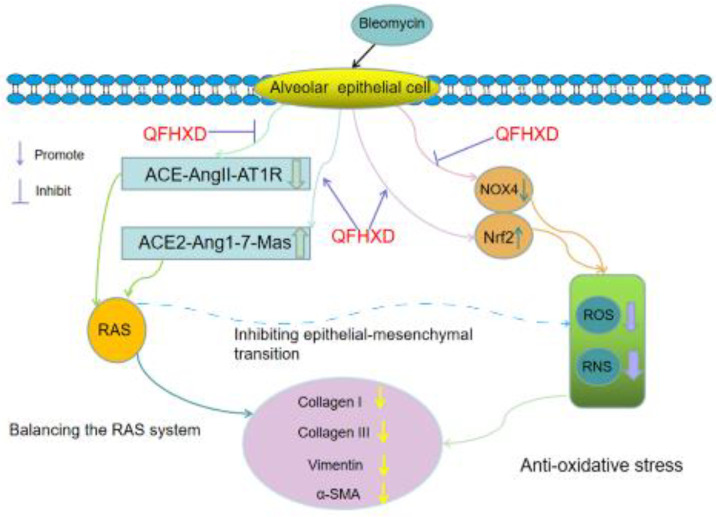
Molecular mechanism diagram of Qing Fei Hua Xian decoction against Bleomycin-induced pulmonary fibrosis

The potential mechanism of QFHXD exerting on PF was shown in Figure 7. As a result of AEC apoptosis and alveolar fibrosis, PF could be induced by BLM. QFHXD may attenuate collagen deposition and marker protein expressions to further protect PF by suppressing oxidative stress by balancing the RAS system.

## Discussion

There are obvious advantages of Traditional Chinese Medicine (TCM) in the process of treating PF, due to its rich experience (21). A previous study has shown that the Jinshui Huanxian formula (JHF) could suppress oxidative stress by restoring the balance of Nrf2-NOX4 in the treatment of PF (22). QFHXD, a Chinese medicine formula constructed based on the characteristics of PF patients has been proven effective in alleviating clinical symptoms and relieving inflammatory response and fibrosis deposition. In this study, BLM was used to establish the PF rat models. QFHXD was administered to BLM-induced PF rats at 413.3, 826.6, and 1239.9 mg/kg doses to determine its therapeutic effect. This study found that QFHXD significantly down-regulated the ACE-AngII-AT1R axis and up-regulated the ACE2-Ang-(1-7)-Mas axis, achieving the RAS system balance, subsequently exhibiting anti-oxidative stress property via lowering NOX4 level and facilitating Nrf2 growth in lung tissues. Additionally, QFHXD suppressed alveolus inflammation, collagen protein expressions, vimentin, and α-SMA expressions to inhibit PF. 

PF is a chronic lung disease characterized by inflammation of lung tissues. When collagen synthesis and degradation are imbalanced, fibrosis is characterized by an increase in collagen fragments, which may have pro-inflammatory effects (23, 24). Therefore, PF can be ameliorated by inhibiting alveolar inflammation and inflammatory responses. Using H&E staining, we observed alveolar structure disruption and inflammatory cell infiltration in the fibrosis tissues. The alveolitis was ameliorated after administration with a 1239.9 mg/kg dose of QFHXD. Additionally, QFHXD significantly attenuated the fibrosis deposition in groups receiving 826.6 and 1239.9 mg/kg doses of QFHXD with MT staining. It can be speculated that QFHXD could reduce alveolar inflammation and fibrosis deposition, thus exerting a therapeutic effect on PF. 

Alveolar epithelium injury and abnormal repair are critical for PF initiation (25). ECM, which consists of fibrillar collagen and fibronectin, promotes alveolar epithelial type II cell (AT2) regeneration failure in lung injury repair (26). The major ECM proteins (collagen and elastin) serve as scaffolding for cells and tissues. During wound healing and fibrosis, collagen I is synthesized initially and at a quicker rate than collagen III. As a consequence, the composition of the ECM can affect the stiffness of lung tissues due to the different ratios of collagen I to III (27, 28). In addition, large quantities of ECM proteins, such as collagen I and extra domain A (EDA) fibronectin, are secreted by α-SMA, whose deposition is crucial in the process of wound repair (29). A previous study has suggested that vimentin could coordinate important cellular activities that control wound healing, such as collagen accumulation (30). Herein, a QFHXD treatment decreased the expression of ECM proteins and fibrosis-linked proteins in lung tissues of rats induced by BLM. QFHXD administration attenuated these protein expressions in a dose-dependent manner. These results suggested that QFHXD may have an anti-fibrotic effect partly by attenuating ECM deposition.

Additionally, AT2 injury/dysfunction and even death can lead to loss of lung function and contribute to IPF (31, 32). The RAS system is made up of ACE-AngII-AT1R and ACE2-Ang-(1-7)-Mas axes. Important consequences for early PF development and progression are discovered to be associated with imbalances in these two axes. During BLM-induced PF, elevated levels of ACE, AngII, and AT1R were strongly associated with disease progression (33). Nevertheless, overexpression of ACE2 and Ang-(1-7) may protect against BLM- or AngII-induced PF by suppressing the MAPK/NF-κB pathway (12). In this study, QFHXD up-regulated ACE2 and Mas expression levels, while correspondingly weakening the expression levels of ACE, AGT, and AT1R. Intratracheal injection of AGT antisense oligonucleotide (ASO) has been shown to inhibit AGT synthesis, AEC death, and collagen accumulation in BLM-induced PF Wistar rats (34). The results of this study revealed that QFHXD balanced the RAS system by switching the ACE-AngII-AT1R to the ACE2-Ang-(1-7)-Mas axis, which helped stop the progression of PF.

BLM-induced PF appears to result from an inflammatory lesion accompanied by a macrophage and neutrophil accumulation in the lower respiratory tract (35). In this lesion, activated inflammatory cells may accumulate and release harmful amounts of ROS. This may be involved in parenchymal injury and alveolar fibrosis (36). Both overexpression of NOX4 and low expression of Nrf2 lead to impaired redox homeostasis, which contributes to oxidative stress associated with PF metabolism (37). NOX4 plays a crucial role in regulating intracellular ROS production. Thus, inhibition of NOX4 activity to regulate intracellular ROS generation has become a major therapeutic approach to treating fibrosis (38). Moreover, in the BLM-induced PF model, Nrf2 regulates anti-oxidant production and defense enzyme expression, thereby protecting PF against oxidative damage (39). In the present study, QFHXD administration achieved Nrf2-NOX4 redox homeostasis by down-regulating NOX4 protein expression and up-regulating the Nrf2 protein level, which had a protective effect against lung injury and PF. An earlier study has demonstrated that alveolar injury, α-SMA up-regulation, and ECM component secretion and deposition were NOX4-dependent in a BLM-induced PF model and that NOX4 inhibitors markedly reduced the established fibrotic response (40). Nrf2 expression is negatively correlated with α-SMA and collagen I expression, and thus activating Nrf2 promotes anti-oxidant defense against IPF (41). According to the findings in this study, QFHXD may reduce alveolar injury and collagen deposition, as well as marker protein expressions, by inhibiting NOX4 expression and facilitating Nrf2 expression. 

Additionally, AngII-induced NOX-dependent superoxide activation is a major pathogenic factor in PF progression (42). Yue *et al*. found that hyperoxic lung injury oxidative stress was attenuated through ACE2 regulation of the Nrf2 pathway as well as its downstream anti-oxidant enzymes (43). These observations first revealed that QFHXD exerted anti-oxidant effects by up-regulating the ACE2-Ang-(1-7)-Mas axis and down-regulating AngII expression, as well as the ACE-AngII-AT1R axis to delay PF progression. Thus, it was speculated that QFHXD may further protect PF by inhibiting oxidative stress by balancing the RAS system. Nevertheless, it is still unclear how QFHXD inhibits oxidative stress by balancing these two axes and thus reduces fibrosis. Therefore, the underlying mechanism needs to be further investigated. 

There are some limitations to this study. As QFHXD was superior to prednisone in anti-fibrosis studies for PF animal models, further studies will be necessary to speculate on the influence of QFHXD in treating PF patients clinically due to the difference between animal models and human PF patients. This study was limited to the lack of inflammatory factors and further signaling pathway studies. 

## Conclusion

This study revealed that as a constructed Chinese medicine formula, QFHXD, was effective at suppressing inflammation-induced infiltration of lung tissues and PF by reducing oxidative stress through restoring the balance of ACE-AngII-AT1R and ACE2-Ang-(1-7)-Mas axes. The findings suggested that QFHXD might be a viable clinical therapy for PF.

## Authors’ Contributions

CW, HK, XW, and YZ Conceived the study and design; RL Performed data processing, collection, experiments, analysis and interpretation of results, and visualization, and prepared the draft manuscript; YZ Critically revised or edited the article; CW, HK, and XW Supervised the research; RL, CW, HK, XW, and YZ Approved the final version to be published.

## Conflicts of Interest

The authors declare that there are no conflicts of interest regarding the publication of this article. 

## References

[1] Parimon T, Hohmann MS, Yao C (2021). Cellular Senescence: Pathogenic mechanisms in lung fibrosis. Int J Mol Sci.

[2] Raghu G, Collard HR, Egan JJ, Martinez FJ, Behr J, Brown KK (2011). An official ATS/ERS/JRS/ALAT statement: idiopathic pulmonary fibrosis: evidence-based guidelines for diagnosis and management. Am J Respir Crit Care Med.

[3] Lederer DJ, Martinez FJ (2018). Idiopathic Pulmonary Fibrosis. N Engl J Med.

[4] Wang D, Yan Z, Bu L, An C, Deng B, Zhang J (2019). Protective effect of peptide DR8 on bleomycin-induced pulmonary fibrosis by regulating the TGF-beta/MAPK signaling pathway and oxidative stress. Toxicol Appl Pharmacol.

[5] Kato K, Hecker L (2020). NADPH oxidases: Pathophysiology and therapeutic potential in age-associated pulmonary fibrosis. Redox Biol.

[6] Hecker L, Cheng J, Thannickal VJ (2012). Targeting NOX enzymes in pulmonary fibrosis. Cell Mol Life Sci.

[7] Hecker L, Vittal R, Jones T, Jagirdar R, Luckhardt TR, Horowitz JC (2009). NADPH oxidase-4 mediates myofibroblast activation and fibrogenic responses to lung injury. Nat Med.

[8] Cheresh P, Kim SJ, Tulasiram S, Kamp DW (2013). Oxidative stress and pulmonary fibrosis. Biochim Biophys Acta.

[9] Zhang Z, Qu J, Zheng C, Zhang P, Zhou W, Cui W (2018). Nrf2 anti-oxidant pathway suppresses Numb-mediated epithelial-mesenchymal transition during pulmonary fibrosis. Cell Death Dis.

[10] Peng LY, An L, Sun NY, Ma Y, Zhang XW, Liu WH (2019). Salvia miltiorrhiza Restrains Reactive Oxygen Species-Associated Pulmonary Fibrosis via Targeting Nrf2-Nox4 Redox Balance. Am J Chin Med.

[11] Shenoy V, Ferreira AJ, Qi Y, Fraga-Silva RA, Diez-Freire C, Dooies A (2010). The angiotensin-converting enzyme 2/angiogenesis-(1-7)/Mas axis confers cardiopulmonary protection against lung fibrosis and pulmonary hypertension. Am J Respir Crit Care Med.

[12] Meng Y, Yu CH, Li W, Li T, Luo W, Huang S (2014). Angiotensin-converting enzyme 2/angiotensin-(1-7)/Mas axis protects against lung fibrosis by inhibiting the MAPK/NF-kappaB pathway. Am J Respir Cell Mol Biol.

[13] Meng Y, Li T, Zhou GS, Chen Y, Yu CH, Pang MX (2015). The angiotensin-converting enzyme 2/angiotensin (1-7)/Mas axis protects against lung fibroblast migration and lung fibrosis by inhibiting the NOX4-derived ROS-mediated RhoA/Rho kinase pathway. Anti-oxid Redox Signal.

[14] Qian W, Cai X, Qian Q, Wang D, Zhang L (2020). Angelica Sinensis Polysaccharide Suppresses Epithelial-Mesenchymal Transition and Pulmonary Fibrosis via a DANCR/AUF-1/FOXO3 Regulatory Axis. Aging Dis.

[15] Wang Z, Fang K, Wang G, Guan X, Pang Z, Guo Y (2019). Protective effect of amygdalin on epithelial-mesenchymal transformation in experimental chronic obstructive pulmonary disease mice. Phytother Res.

[16] Shi W, Hao J, Wu Y, Liu C, Shimizu K, Li R (2022). Protective effects of heterophyllin B against bleomycin-induced pulmonary fibrosis in mice via AMPK activation. Eur J Pharmacol.

[17] Luo C, Wang H, Chen X, Cui Y, Li H, Long J (2013). Protection of H9c2 rat cardiomyoblasts against oxidative insults by total paeony glucosides from Radix Paeoniae Rubrae. Phytomedicine.

[18] Szapiel SV, Elson NA, Fulmer JD, Hunninghake GW, Crystal RG (1979). Bleomycin-induced interstitial pulmonary disease in the nude, athymic mouse. Am Rev Respir Dis.

[19] Xu Y, Hang WL, Zhou XM, Wu Q (2021). Exploring the mechanism whereby sinensetin delays the progression of pulmonary fibrosis based on network pharmacology and pulmonary fibrosis models. Front Pharmacol.

[20] Li X, Zhuang J, Rayford H, Zhang H, Shu R, Uhal BD (2007). Attenuation of bleomycin-induced pulmonary fibrosis by intratracheal administration of antisense oligonucleotides against angiotensinogen mRNA. Curr Pharm Des.

[21] Zhang Y, Lu P, Qin H, Zhang Y, Sun X, Song X (2021). Traditional Chinese medicine combined with pulmonary drug delivery system and idiopathic pulmonary fibrosis: Rationale and therapeutic potential. Biomed Pharmacother.

[22] Bai Y, Li J, Zhao P, Li Y, Li M, Feng S (2018). A chinese herbal formula ameliorates pulmonary fibrosis by inhibiting oxidative stress via upregulating Nrf2. Front Pharmacol.

[23] McKleroy W, Lee TH, Atabai K (2013). Always cleave up your mess: targeting collagen degradation to treat tissue fibrosis. Am J Physiol Lung Cell Mol Physiol.

[24] Øya E, Becher R, Ekeren L, Afanou AKJ, Øvrevik J, Holme JA (2019). Pro-inflammatory responses in human bronchial epithelial cells induced by spores and hyphal fragments of common damp indoor molds. Int J Environ Res Public Health.

[25] Hewlett JC, Kropski JA, Blackwell TS (2018). Idiopathic pulmonary fibrosis: Epithelial-mesenchymal interactions and emerging therapeutic targets. Matrix Biol.

[26] Chanda D, Otoupalova E, Smith SR, Volckaert T, De Langhe SP, Thannickal VJ (2019). Developmental pathways in the pathogenesis of lung fibrosis. Mol Aspects Med.

[27] Hansen NU, Karsdal MA, Brockbank S, Cruwys S, Rønnow S, Leeming DJ (2016). Tissue turnover of collagen type I, III and elastin is elevated in the PCLS model of IPF and can be restored back to vehicle levels using a phosphodiesterase inhibitor. Respir Res.

[28] Liu G, Philp AM, Corte T, Travis MA, Schilter H, Hansbro NG (2021). Therapeutic targets in lung tissue remodelling and fibrosis. Pharmacol Ther.

[29] Aboonabi A, Singh I (2015). Chemopreventive role of anthocyanins in atherosclerosis via activation of Nrf2-ARE as an indicator and modulator of redox. Biomed Pharmacother.

[30] Cheng F, Shen Y, Mohanasundaram P, Lindstrom M, Ivaska J, Ny T (2016). Vimentin coordinates fibroblast proliferation and keratinocyte differentiation in wound healing via TGF-beta-Slug signaling. Proc Natl Acad Sci U S A.

[31] Parimon T, Yao C, Stripp BR, Noble PW, Chen P (2020). Alveolar epithelial type II cells as drivers of lung fibrosis in idiopathic pulmonary fibrosis. Int J Mol Sci.

[32] Selman M, Pardo A (2020). The leading role of epithelial cells in the pathogenesis of idiopathic pulmonary fibrosis. Cell Signal.

[33] Marshall RP, McAnulty RJ, Laurent GJ (2000). Angiotensin II is mitogenic for human lung fibroblasts via activation of the type 1 receptor. Am J Respir Crit Care Med.

[34] Wu CH, Wang Y, Ma M, Mullick AE, Crooke RM, Graham MJ (2019). Antisense oligonucleotides targeting angiotensinogen: Insights from animal studies. Biosci Rep.

[35] Crystal RG, Bitterman PB, Rennard SI, Hance AJ, Keogh BA (1984). Interstitial lung diseases of unknown cause Disorders characterized by chronic inflammation of the lower respiratory tract. N Engl J Med.

[36] Serrano-Mollar A, Closa D, Prats N, Blesa S, Martinez-Losa M, Cortijo J (2003). In vivo anti-oxidant treatment protects against bleomycin-induced lung damage in rats. Br J Pharmacol.

[37] Hecker L, Logsdon NJ, Kurundkar D, Kurundkar A, Bernard K, Hock T (2014). Reversal of persistent fibrosis in aging by targeting Nox4-Nrf2 redox imbalance. Sci Transl Med.

[38] Stock CJW, Michaeloudes C, Leoni P, Durham AL, Mumby S, Wells AU (2019). Bromodomain and extraterminal (BET) protein inhibition restores redox balance and inhibits myofibroblast activation. Biomed Res Int.

[39] Walters DM, Cho HY, Kleeberger SR (2008). Oxidative stress and anti-oxidants in the pathogenesis of pulmonary fibrosis: a potential role for Nrf2. Anti-oxid Redox Signal.

[40] Jarman ER, Khambata VS, Cope C, Jones P, Roger J, Ye LY (2014). An inhibitor of NADPH oxidase-4 attenuates established pulmonary fibrosis in a rodent disease model. Am J Respir Cell Mol Biol.

[41] Artaud-Macari E, Goven D, Brayer S, Hamimi A, Besnard V, Marchal-Somme J (2013). Nuclear factor erythroid 2-related factor 2 nuclear translocation induces myofibroblastic dedifferentiation in idiopathic pulmonary fibrosis. Anti-oxid Redox Signal.

[42] Yang J, Tan Y, Zhao F, Ma Z, Wang Y, Zheng S (2011). Angiotensin II plays a critical role in diabetic pulmonary fibrosis most likely via activation of NADPH oxidase-mediated nitrosative damage. Am J Physiol Endocrinol Metab.

[43] Fang Y, Gao F, Liu Z (2019). Angiotensin-converting enzyme 2 attenuates inflammatory response and oxidative stress in hyperoxic lung injury by regulating NF-kappaB and Nrf2 pathways. QJM.

